# Isolation of human intrahepatic leukocytes for phenotypic and functional characterization by flow cytometry

**DOI:** 10.1016/j.xpro.2022.101356

**Published:** 2022-04-29

**Authors:** Stephanie Kucykowicz, Oliver E. Amin, Alice R. Burton, Leo Swadling, Nathalie M. Schmidt, Nekisa Zakeri, Jessica Davies, Gloryanne Aidoo-Micah, Kerstin A. Stegmann, Nicholas J. Easom, Anna Jeffery-Smith, Mala K. Maini, Laura J. Pallett

**Affiliations:** 1 Institute of Immunity and Transplantation, Division of Infection & Immunity, University College London, London, UK

**Keywords:** Cell isolation, Single Cell, Flow Cytometry/Mass Cytometry, Health Sciences, Immunology

## Abstract

With the growing appreciation of tissue-resident immunity, studying tissue-specific immune cells contributing to both homeostasis and disease is imperative. Here, we provide a protocol for the isolation of human intrahepatic leukocytes (IHL) maximizing viability, purity, and yield. Our protocol is scalable by tissue weight, allowing for reproducible and efficient IHL liberation suitable for functional characterization, cell isolation, and profiling by flow (or mass) cytometry. Furthermore, we provide a “guide” to determine an expected IHL yield per gram of tissue processed.

For complete details on the use and execution of this protocol, please refer to Stegmann et al. (2016), Pallett et al. (2017), Easom et al. (2018), Swadling et al. (2020), Pallett et al. (2020), and Zakeri et al. (2022).

## Highlights

• Improved protocol for the efficient and reproducible isolation of intrahepatic leukocytes
• Comprehensive protocol combining enzymatic, mechanical, and density gradient centrifugation
• Detailed guide for characterization of a single IHL suspension by flow (mass) cytometry
• Tool to predict intrahepatic leukocyte yield per gram of tissue processed

## Graphical abstract

**Figure:**
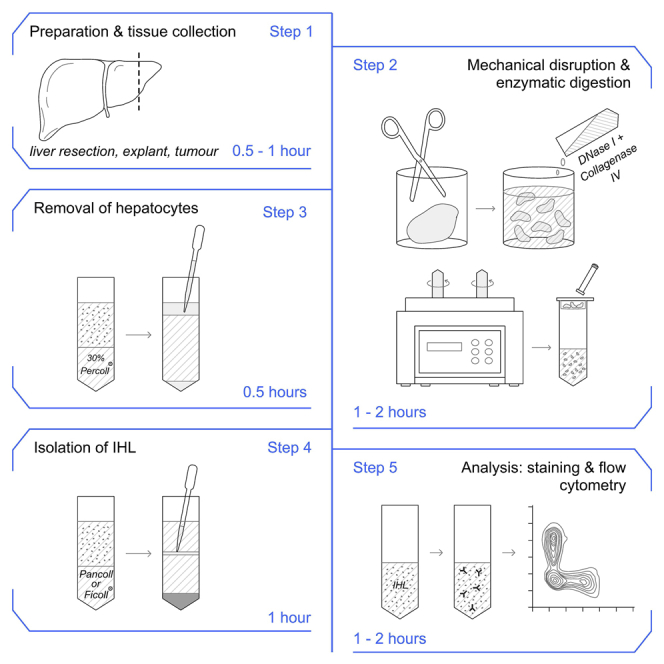

## Before you begin

The protocol below describes the specific steps for the efficient isolation of intrahepatic leukocytes (IHL) from human liver tissue of variable size and weight including those obtained from resections and explant surgeries. The protocol is highly reproducible and can be scaled as required depending on the size and weight of liver sample available. For optimal results, tissue samples should be stored immediately in University of Wisconsin Solution (or RPMI-1640 media if unavailable; refer to key resources table) and processed within 6 h of surgical retrieval. If not processed immediately after surgical removal from the patient, samples should be stored at 4°C prior to processing, which we would recommend being limited to less than 24 h (older samples have not been tested).

Isolated IHL can be used for short-term primary cell culture or phenotypic and functional analyses *ex vivo*. Investigators should be proficient with aseptic cell culture, multi-parameter flow cytometry (e.g., the use of a BD Bioscience LSRFortessa X20) and data analysis (e.g., the use of FlowJo). Here we focus on the identification of lymphocyte subsets, but this protocol can be adapted to characterize cell populations of myeloid lineage, or for the isolation and analysis of tumor infiltrating lymphocytes (TIL) from liver tumors. Where sufficient quantity of tissue allows, this protocol can also be adapted to enable the isolation of liver-resident macrophages (Kupffer cells [KC]) and hepatic stellate cells (HSC); however, this will require modification of density gradients as described in detail in a previously published article (Cossarizza et al., 2021).

### Institutional permissions

All samples used in the optimization of this protocol were obtained with regulatory oversight from the *Tissue Access for Patient Benefit Initiative* at the Royal Free Hospital (RFH) (London; UK), approved by the University College London (UCL) RFH BioBank Ethical Review Committee (UCL/RFH BioBank; REC reference: 11/WA/0077 or 21/WA/0388).

Appropriate ethical approval from your relevant institutions is required to process and undertake experiments using patient-derived materials with this protocol.

### Preparation of reagents

**Timing: 0.5–1 h**

1. Enzyme digestion stocks (DNase I & Collagenase IV)∗.
2. Pre-warmed media (1× HBSS with calcium and magnesium chloride [HBSS^+/+^], 1× HBSS without calcium and magnesium chloride [HBSS^−/−^] & RPMI-1640) - 37°C.
3. Enzyme digestion buffer (0.002% DNase I & 0.02% Collagenase IV).
4. 30% Percoll®.
5. LIVE/DEAD™ Fixable Blue Dead Cell Stain.
6. Freezing media (90% heat-inactivated fetal bovine serum (Hi-FBS) + 10% DMSO)∗.

***Note:*** ∗Aliquots of these reagents can be made in advance and stored at −20°C for up to 1 year to reduce the time spent preparing reagents when performing this protocol each time.

### Preparation of equipment

**Timing: 0.5 h**

7. Switch on Class II Microbiological Safety Cabinet (MSC).
8. Sterilize equipment (blunt-ended scissors and forceps) and preparation area within MSC with 70% ethanol (or equivalent).

***Note:*** Equipment sterilization is only necessary if IHL will be used for culture.

## Key resources table

**Table:** 

REAGENT or RESOURCE	SOURCE	IDENTIFIER
**Antibodies**		

Anti-human CD3 PE-Cy7 (clone OKT3) 1:100	BioLegend	Cat# 317334
Anti-human CD45 BUV805 (clone HI30) 1:100	BD Biosciences	Cat# 557871
Anti-human CD19 BV786 (clone HIB19) 1:100	BD Biosciences	Cat# 740968
Anti-human CD56 PE/Dazzle 594 (clone QA17A16) 1:100	BioLegend	Cat# 392410

**Biological samples**		

Liver tissue (background liver and tumor) from patients undergoing diagnostic tests, transplantation (explant) or surgical resection for various diagnoses	Tissue Access for Patient Benefit (TAPb)	https://www.ucl.ac.uk/tapb/

**Chemicals, peptides, and recombinant proteins**		

DNase I	Roche	Cat# 11284932001
Collagenase IV	Thermo Fisher Scientific	Cat# 17104-019
1× Hanks’ balanced salt solution - HBSS^+/+^ (with Ca^2+^ and Mg^2+^)	Thermo Fisher Scientific	Cat# 24020-091
1× Hanks’ balanced salt solution - HBSS^−/−^(without Ca^2+^ and Mg^2+^)	Thermo Fisher Scientific	Cat# 14170-088
Pancoll	PAN-Biotech	Cat# P04-60500
Percoll®	VWR	Cat# 17-0891-02
Dimethyl sulfoxide (DMSO)	Sigma-Aldrich	Cat# D2650-100ml
1× Phosphate-buffered saline (PBS)	Thermo Fisher Scientific	Cat# 14190169
1× Roswell Park Memorial Institute 1640 medium (RPMI-1640)	Thermo Fisher Scientific	Cat# 21875091
University of Wisconsin cold storage solution (Belzer UW)	Bridge to Life (Europe)	n/a
Ethanol	Various	Various
Heat-inactivated fetal bovine serum (Hi-FBS)	Sigma-Aldrich	Cat# F9665
Cytofix^TM^	BD Biosciences	Cat# 554655
Brilliant stain buffer	BD Biosciences	Cat# 563794
FcR blocking reagent	Miltenyi Biotec	Cat# 130-059-901
Red blood cell lysis buffer (10×)	BioLegend	Cat# 420301

**Critical commercial assays**		

LIVE/DEAD^TM^ Fixable Blue Dead Cell Stain Kit	Thermo Fisher Scientific	Cat# 2745275

**Software and algorithms**		

FlowJo v. 10	BD Biosciences	https://www.flowjo.com/
BD FACSDiva Software v. 9.0.1	BD Biosciences	n/a

**Other**		

Blunt-ended scissors	VWR	Cat# 2650780
LSRFortessa X20 flow cytometer	BD Biosciences	n/a
Forceps	VWR	Cat# 613-5074
Centrifuge	Eppendorf	Various
Mr. Frosty^TM^ freezing container	Thermo Fisher Scientific	Cat# 5100-0001
gentleMACS^TM^ C-tubes	Miltenyi Biotec	Cat# 130-096-334
100 mL multipurpose plastic beaker	Greiner	Cat# 724461
70 μm cell strainer	Greiner	Cat# 542070
Sterile 5 mL syringe	VWR	Cat# 613-2043
25 mL serological pipettes	Greiner	Cat# 760180
50 mL tubes	Greiner	Cat# 227285
Sterile Pasteur pipettes	Greiner	Cat# 612398
Cryovials	Greiner	Cat# 123277
FACS tubes	Sarstedt	Cat# 55.1579
96-well plate U-bottom	Sarstedt	Cat# 83.3925.500
ADAM-MC^TM^ counting chambers (or equivalent)	Science Services GmbH	Cat# AD4K-200

## Materials and equipment

### Enzyme digestion buffer

Prepare enzyme digestion stocks by dissolving 100 mg of Collagenase IV or DNase I in 10 mL HBSS^+/+^ to obtain working concentrations (50× and 500× final concentration respectively). Once in solution, sterilize by filtering through a 0.45 μm filter and freeze in individual well-labeled aliquots. Store enzyme stocks at −20°C for up to 1 year. Date aliquots prior to freezing.

**Table:** Enzyme preparation

Reagent	Working stock concentration	Activity	Quantity	Volume HBSS^+/+^
Collagenase IV	50× (1%)	>160 units/mg	100 mg	10 mL
DNase I	500× (1%)	>2,000 units/mg	100 mg	10 mL

**CRITICAL:** To avoid freeze/thaw cycles of enzyme stocks, aliquot in volumes needed for preparation of 50 mL digestion buffer i.e., 1 mL aliquots for Collagenase IV and 100 μL aliquots for DNase I, as per table below.

Dilute the 50× Collagenase IV and 500× DNase I working stocks in 1× HBSS^+/+^ to achieve an appropriate final working concentration of 0.02% and 0.002% respectively, as shown in table below. Incubate tubes with complete enzyme digestion buffer in a water bath pre-warmed to 37°C prior to tissue digestion.

**Table:** Enzyme digestion buffer

Reagent	Final concentration	Amount
50× collagenase IV stock	1× (0.02%)	1 mL
500× DNase I stock	1× (0.002%)	100 μL
1× HBSS^+/+^	n/a	48.9 mL
**Total**	**n/a**	**50 mL**

Prepare enzyme digestion buffer fresh prior to tissue processing.

**CRITICAL:** Prepare appropriate volume of enzyme digestion buffer in advance. We suggest 50 mL of enzyme digestion buffer for every 30 g of liver tissue (equivalent to ∼3.5 × 3.5 × 3.5 cm). Ensure the HBSS used contains calcium (Ca^2+^) and magnesium (Mg^2+^) to support enzymatic activity of the Collagenase IV and DNase I. For maximal enzymatic digestion, allow enzyme digestion buffer to reach 37°C prior to use.

### 30% Percoll®

A solution of 30% Percoll®, prepared in 1× HBSS^−/−^, is used for the differential density gradient to remove contaminating parenchymal cells, including dead or dying hepatocytes. Ensure use of HBSS^−/−^ media for Percoll® preparation to stop any lasting enzymatic digestion and prevent any residual activity resulting in cleavage of surface proteins from cells of interest. Prepare the 30% Percoll fresh prior to tissue processing and ensure solution is at room temperature.

**Table:** 30% Percoll

Reagent	Final concentration	Amount
Percoll®	30% (v/v)	15 mL
1× HBSS^−/−^	70% (v/v)	35 mL
**Total**	**n/a**	**50 mL**

### LIVE/DEAD™ Fixable Blue Dead Cell Stain

**CRITICAL:** When staining IHL, we recommend using an appropriate marker of cell viability. We have optimized our protocol for use with the LIVE/DEAD^TM^ Fixable Blue Dead Cell Stain. Alternative reagents for measuring cell viability using different fluorochrome combinations are available (refer to options below or to alternative resources table). These will require titration and optimization.

Reconstitute 1 vial of lyophilized LIVE/DEAD™ Fixable Blue Dead Cell Stain with 50 μL of DMSO as per manufacturer’s instructions. Vortex the sample immediately to mix. Spin contents and freeze at −20°C protected from light. We recommend a 1,000× dilution in 1× PBS for optimal staining and dead cell detection. Make smaller aliquots of the stock solution to reduce freeze-thaw cycles. LIVE/DEAD™ Fixable Blue Dead Cell Stain has an excitation maximum of ∼350 nm, making it ideal for use with a UV laser, and an emission of ∼450 nm.

***Alternatives:*** Other viability dyes are available from the same manufacturer based on different excitation/emission spectra (e.g., LIVE/DEAD^TM^ Fixable Violet Stain and LIVE/DEAD^TM^ Fixable Near-IR Stain; refer to alternative resources table) and from other manufacturers (e.g., fixable Zombie^TM^ dyes from BioLegend) that are compatible with this protocol.

### Cell freezing media

Prepare 10% DMSO (v/v) solution in heat-inactivated FBS (Hi-FBS) and make 50 mL aliquots. Store aliquots at 4°C for up to 2 months or at −20°C for up to 1 year.

**Table:** Cell freezing media

Reagent	Final concentration	Amount
Hi-FBS	90% (v/v)	45 mL
DMSO	10% (v/v)	5 mL
**Total**	**n/a**	**50 mL**

### BD LSRFortessa X20 flow cytometer

The example antibody panel detailed in this protocol (refer to key resources table and staining of leukocytes for flow cytometry protocol) was designed to be run on a 5 laser BD LSRFortessa X20 flow cytometer with the configuration detailed in the table below. Depending on the instrument configuration available to you, alternative antibody and fluorochrome combinations may be required to probe your cell(s) of interest.

This cytometer configuration will enable the detection of a panel of antibodies using ∼16 fluorochromes. Thus, the example panel detailed in the staining section of this protocol can serve as a backbone for identification of basic lymphocyte populations (e.g., T cells, B cells, NK cells and NKT cells) with antibodies optimally titrated. However, if necessary, alternative antibodies and fluorochrome combinations can be used to further characterize different isolated leukocyte populations.

**Table:** 

Laser	LP mirror	BP filter
488	685	695/40
488	505	530/30
640	750	780/60
640	790	730/45
640	–	670/14
405	750	780/60
405	685	710/50
405	595	610/20
405	505	525/50
405	–	400/50
355	770	820/60
355	410	450/50
355	–	379/28
561	750	780/60
561	600	610/20
561	–	586/15

### gentleMACS^TM^ tissue dissociator

To follow this protocol in full, a Miltenyi Biotec gentleMACS^TM^ tissue dissociator is required. Furthermore, it requires the gentleMACS^TM^ to be appropriately programmed to ensure access to the pre-set setting *‘m_spleen_01.01’*, for tissue disruption and IHL liberation from samples. To efficiently process multiple samples at one time or to process larger volume samples, we recommend using tissue dissociators capable of processing >2 C-tubes simultaneously.

Alternatively, this protocol can be carried out without using a gentleMACS^TM^ tissue dissociator by manually grinding the tissue or using other commercially available tissue dissociating machines. In this case, you might need to allow more time for tissue processing steps and optimization in your own lab.

### Alternative resources table

**Table:** 

Reagent or resource	Alternative	Company	Catalog number
Anti-human CD3 antibody	Anti-human CD3 BUV395 (clone UCHT1) 1:100	BD Biosciences	Cat# 563546
	Anti-human CD3 PE-CF594 (clone UCHT1) 1:100	BD Biosciences	Cat# 562280
	Anti-human CD3 BV711 (clone OKT3) 1:100	BioLegend	Cat# 317328
	Anti-human CD3 BV605 (clone OKT3) 1:100	BD Biosciences	Cat# 317322
	Anti-human CD3 BV510 (clone OKT3) 1:100	BioLegend	Cat# 317332
Anti-human CD19 antibody	Anti-human CD19 BV510 (clone SJ25C1) 1:100	BioLegend	Cat# 562947
	Anti-human CD19 APC/Cy7 (clone SJ25C1) 1.5:100	BD Biosciences	Cat# 557791
	Anti-human CD19 eFluor450 (clone 2H7) 1:100	BD Biosciences	Cat# 560631
Anti-human CD56 antibody	Anti-human CD56 PE/Cy7 (clone NCAM13.2) 1:100	BD Biosciences	Cat# 335791
	Anti-human CD56 PE/Cy7 (clone NCAM16.2) 1:100	BD Biosciences	Cat# 335826
	Anti-human CD56 PE/Cy7 (clone HCD56) 1:100	BioLegend	Cat# 318317
	Anti-human CD56 ECD (clone N901) 2:100	Beckman Coulter	Cat# AB2943
LIVE/DEAD^TM^ Fixable Blue Dead Cell Stain	LIVE/DEAD^TM^ Fixable Violet Stain	Thermo Fisher Scientific	Cat# L34963
	LIVE/DEAD^TM^ Fixable Near-IR Stain	Thermo Fisher Scientific	Cat# L34975
Pancoll	Ficoll®Paque	Sigma-Aldrich	Cat# 17-1440-03
	Lymphoprep™	STEMCELL Technologies	Cat# 07851
Percoll®	OptiPrep	STEMCELL Technologies	Cat# 07820
DNase I	DNase I	Sigma-Aldrich	Cat# D4527
	DNase I	Thermo Fisher Scientific	Cat# EN052
Collagenase IV	Collagenase IV	Sigma-Aldrich	Cat# CA-22-1G
	Collagenase IV	Cell Signaling Technology	Cat# 44204S
1× HBSS^+/+^	EBSS ^+/+^	Thermo Fisher Scientific	Cat# 24010043
	DPBS ^+/+^	Thermo Fisher Scientific	Cat# 14287080
	HBSS^+/+^	Sigma-Aldrich	Cat# 55037C-1000ML
1× HBSS^−/−^	EBSS^−/−^	Thermo Fisher Scientific	Cat# 14155063
	PBS	Thermo Fisher Scientific	Cat# 14190169
	RPMI-1640	Thermo Fisher Scientific	Cat# 11875093
	DMEM	Thermo Fisher Scientific	Cat# 61965-059
	HBSS^−/−^	Sigma-Aldrich	Cat# 55021C-1000ML
ADAM automated cell counter	Manual cell counting using haemocytometer and trypan blue 0.4%	Sigma-Aldrich	Cat# T8154-100ml
	CASY Cell Counter	Cambridge Bioscience	https://www.bioscience.co.uk/cpl/casy-cell-counter
FlowJo	Kaluza	Beckman Coulter	https://www.beckman.com/flow-cytometry/software/kaluza
	In house analysis scripts	n/a	n/a

## Step-by-step method details

### Mechanical disruption and enzymatic digestion of liver tissue

**Timing: 1–2 h**

**Figure 1:**
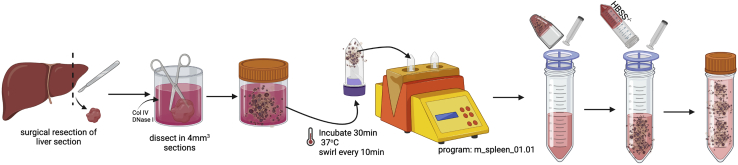
Illustration of the initial steps to mechanically disrupt and enzymatically digest human liver tissue into a single-cell suspension

The following steps provide information on how to dissect, digest and mechanically liberate leukocytes from human liver tissue. The initial steps are illustrated in Figure 1.

Before starting, all reagents and equipment should be prepared in advance to the appropriate volume, concentration and temperature described in this protocol. For optimal results, tissue samples should be processed within 6 h of surgical retrieval, refer to troubleshooting 1 for further details. If not processed immediately after surgical removal from the patient, liver tissue samples should be stored at 4°C in UW solution prior to processing.

**CRITICAL:** Tissue samples must not be allowed to ‘dry out’ at any time during this protocol as this will impact cell viability. Where possible, always dissect tissue in solution.

**Figure 2:**
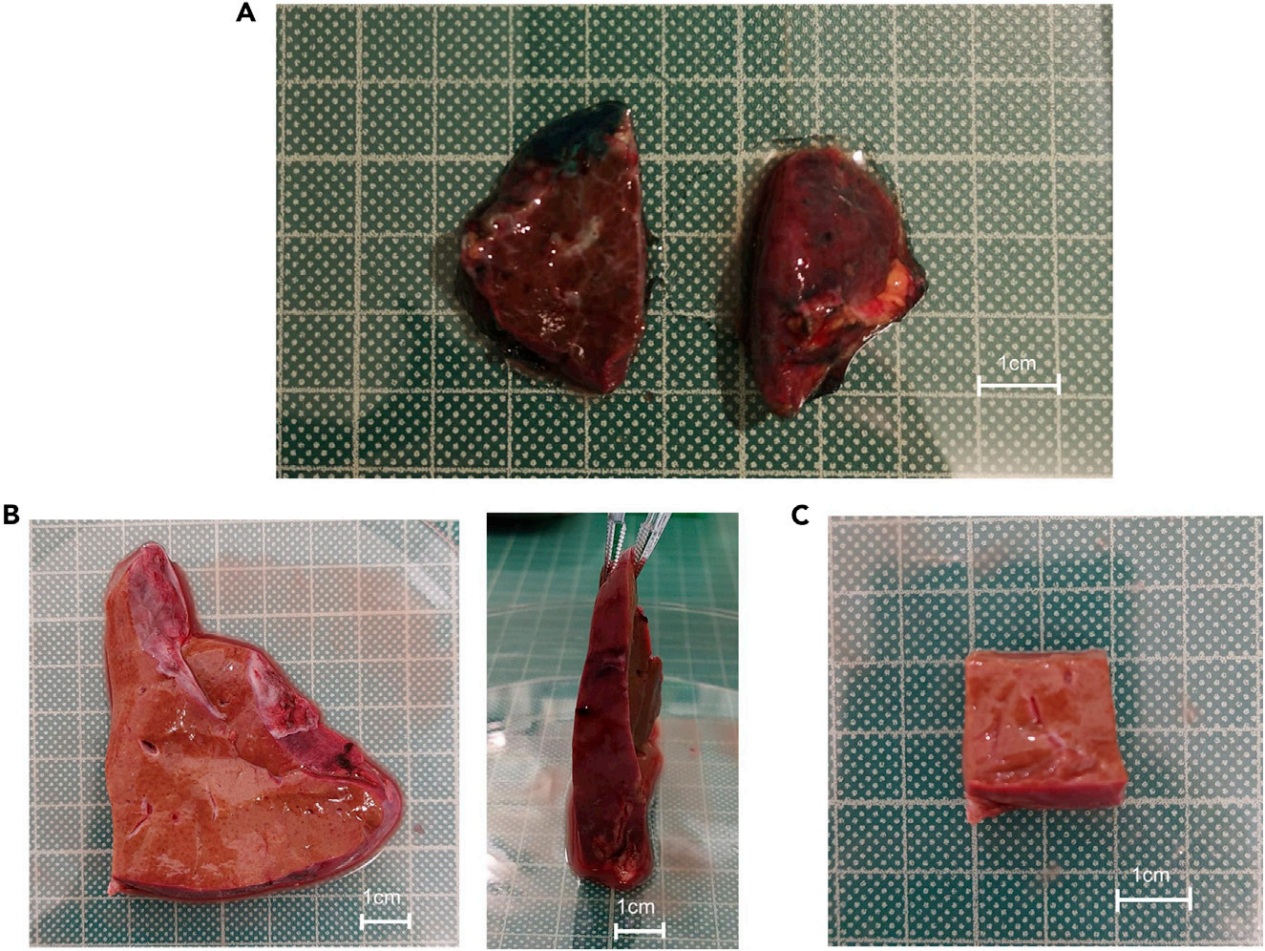
Representative examples of liver resections used for processing (A and B) Example resection weighing. (A) 7.6 g (combined weight) and (B) 22.6 g, shown from 2 different angles. (C) Representative liver resection dissected accurately to represent 6 cm^3^ (2 × 2 × 1.5 cm), weighing 3.6 g.
2. Place each 43 cm^3^ (∼30 g) section of liver into a 100 mL universal beaker and top up with 25 mL pre-warmed enzyme digestion buffer.

***Note:*** If final IHL yield is low, refer to troubleshooting 2.

**Figure 3:**
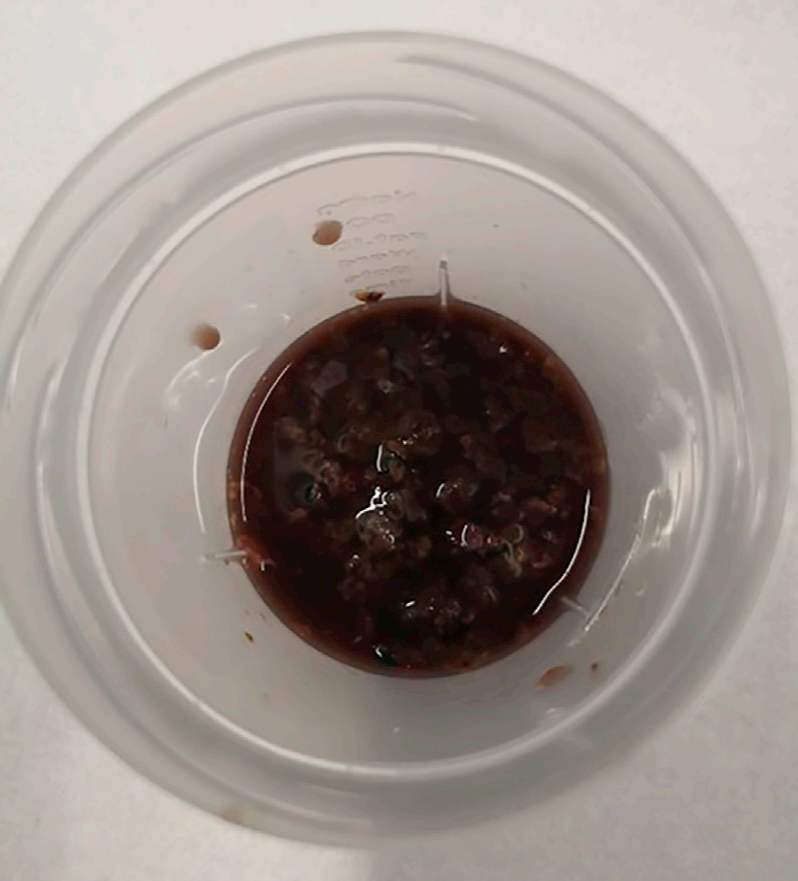
Dissected liver section in small pieces of no greater than 5 × 5 × 5 mm (125 mm^3^) in a 100 mL universal beaker containing pre-warmed enzyme digestion buffer (**step 3**)

1. Remove liver tissue from its preservation solution and dissect, using blunt-ended scissors and forceps, into sections no greater than 3.5 × 3.5 × 3.5 cm (43 cm^3^; max weight: ∼30 g). Reserve preservation solution for **step 5**. Figure 2 is a useful reference guide to liver tissue sizes and corresponding weights.

3. Dissect the larger (43 cm^3^/∼30 g) section of liver into small pieces no greater than 5 × 5 × 5 mm (125 mm^3^) using blunt-ended scissors against the side of the beaker (rough size of pieces depicted in Figure 3). Once chopped, incubate at 37°C for 30 min, swirl the beaker every 10 min.

**CRITICAL:** Tissue must be incubated with enzymes to allow for adequate IHL retrieval. Incubations longer than 30 min may impact surface expression of certain markers. If multiple beakers are being used to process large volumes of tissue, we recommend proceeding to **step 6** one beaker at a time and placing remaining beakers in the fridge after the initial 30 min incubation to stop enzymatic digestion. Alternatively, consider staggering the enzyme incubations to space out the following processing steps.

4. During incubation, place a 70 μm cell strainer on top of a 50 mL conical tube and rinse with 1 mL 1× HBSS^−/−^.
5. Filter strain the liver preservation solution that was set aside in **step 1.** Once filtered reserve until **step 12.**

**CRITICAL:** To maintain aseptic conditions, the following steps need to be carried out in a Class II Microbiological Safety Cabinet (MSC).

6. After 30 min, transfer the enzyme-digested tissue and remaining enzyme digestion buffer into a gentleMACS^TM^ C-tube (not exceeding the maximum volume of 10 mL / C-tube).
7. Place each C-tube on a gentleMACS^TM^ tissue dissociator and run for one cycle using the mouse spleen program: *m_spleen_01.01* (length: 50 s).

***Note:*** If a gentleMACS^TM^ tissue dissociator is unavailable, proceed directly to **step 8**. For sustainability, C-tubes can be re-used after each cycle.

**CRITICAL:** When processing large or multiple samples (size > 43 cm^3^ or weight > 30 g), we recommend staggering the protocol at this point to ensure all timings are adhered to.

**Figure 4:**
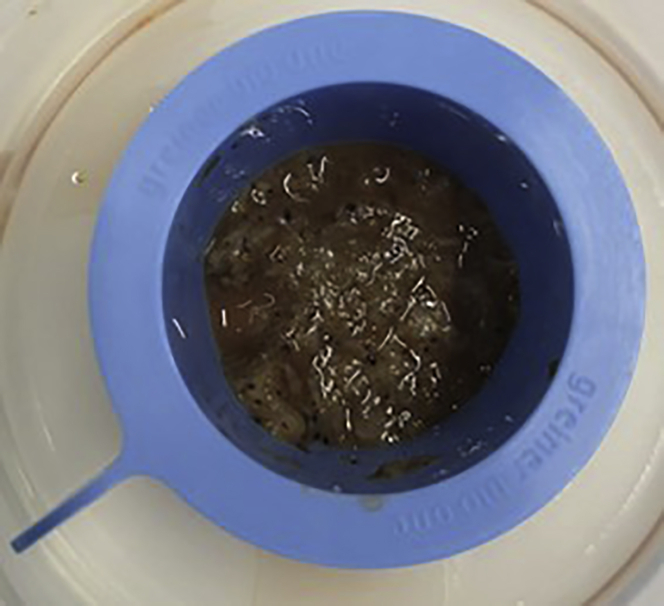
After gentleMACS^TM^ dissociation, the C-tube contents are poured into 70-μm cell strainer placed on top of a 50 mL conical tube and grinded through using the flat end of a sterile syringe plunger and plenty of HBSS^−/−^ (**steps 8–9**)
9. Grind the remaining liver tissue through the cell strainer using the flat end of a sterile plunger from a 5 mL syringe. Wash the cell strainer with 1–2 mL 1× HBSS^−/−^ regularly to prevent clogging and to help ease IHL through (refer to troubleshooting 3).

***Note:*** Take care not to damage the 70 μm cell strainer, replacing as required to prevent blocking of the filter. Discard any tough connective or fibrous tissue that builds up in the cell strainer as necessary.

8. Place a 70 μm cell strainer on a new 50 mL conical tube and rinse with 1 mL 1× HBSS^−/−^. Pour the contents of each C-tube into the cell strainer, as shown in Figure 4. Rinse the cell strainer with 1 mL 1× HBSS^−/−^ before pouring tissue.

10. Once the 50 mL conical tube reaches a volume of 25 mL, remove the cell strainer and top up with a further 25 mL HBSS^−/−^ to prevent over-concentration of cells leading to loss of cell recovery and decreased viability.

***Note:*** Centrifuge cells within 10 min of grinding through a 70 μm cell strainer to prevent further enzymatic digestion.

11. Repeat **steps 6–10** until all tissue is processed and IHL liberated. (If poor final IHL yield, there could be an issue with the tissue drying out or with the amount of time spent in enzyme digestion buffer, refer to troubleshooting 2).

***Note:*** Alternatively, from this point, the cell suspension is suitable for the isolation of HSC and/or KC, rather than IHL, using our previous published protocol. For the full step-by-step method, please refer to Cossarizza et al. (2021)**.**

### Removal of hepatocytes

**Timing: 0.5 h**

**Figure 5:**
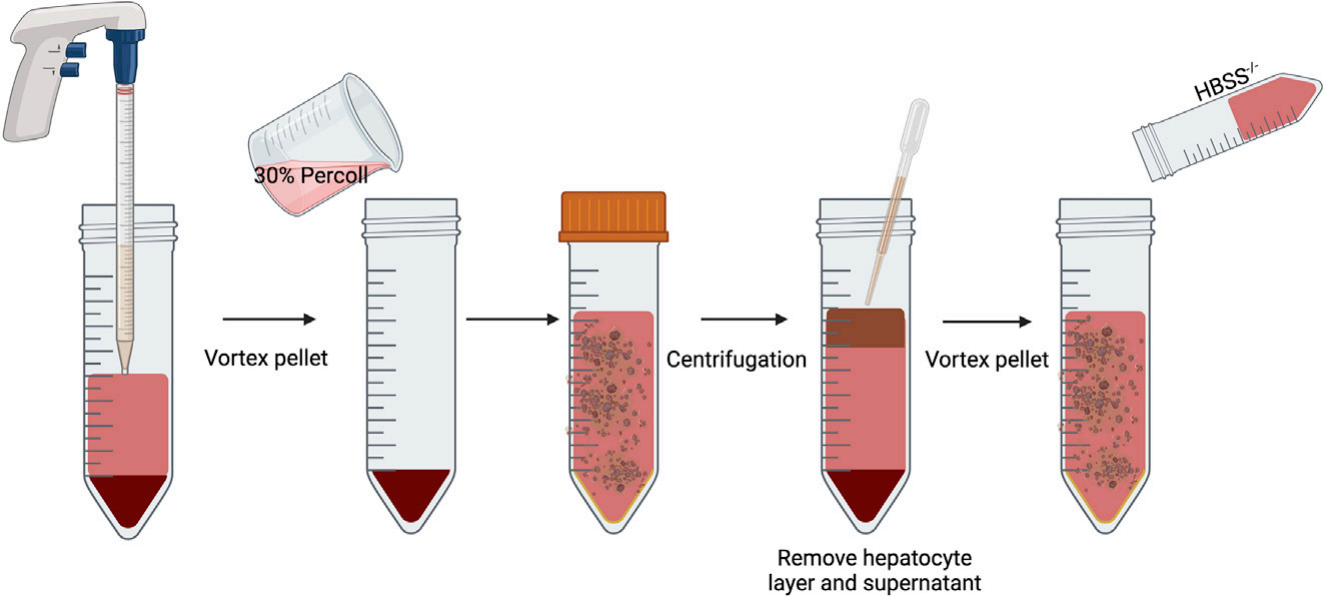
Illustration of the steps required to remove hepatocytes from the initial cell suspension obtained after mechanical and enzymatic digestion of liver tissue

The following steps provide information on how to remove hepatocytes from the cell suspension to enrich for viable IHL. These steps are illustrated in Figure 5. Contaminating hepatocytes will negatively impact the viability of lymphocytes in suspension.

12. Centrifuge the single-cell suspension at 700 g using a swing bucket rotor for 15 min at room temperature (R.T.; acceleration/deceleration: 9/9).
13. Discard the supernatant using a 25 mL serological pipette.

**Figure 6:**
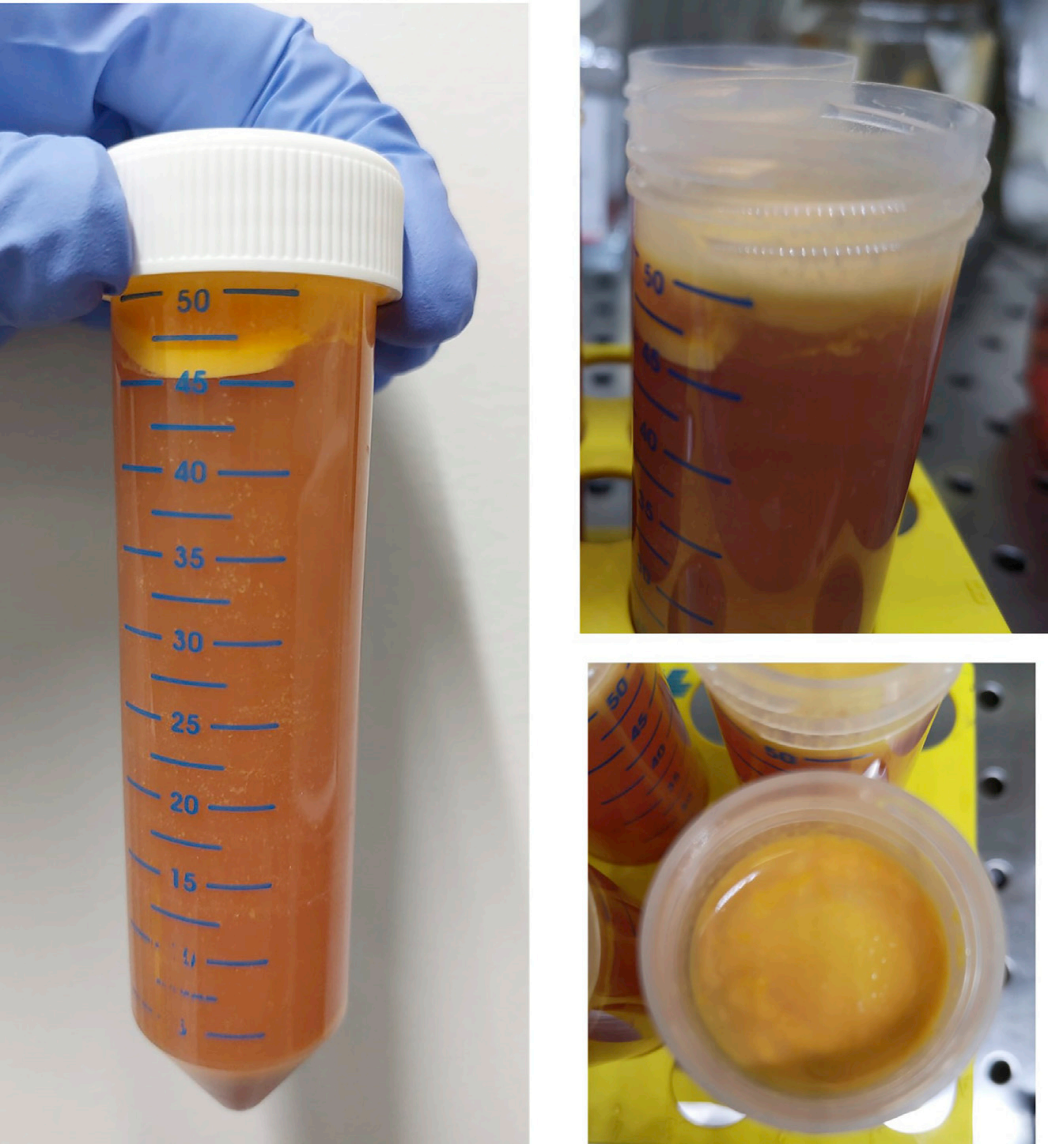
A representative example viewed from different angles of the fat layer floating at the top of the conical tube following centrifugation at **step 12**

***Note:*** Multiple cell pellets can be combined into one 50 mL conical tube before resuspending in 20 mL of 30% Percoll® (v/v) solution following vortexing.

***Note:*** If necessary, first harvest the layer of fat (as illustrated in Figure 6) floating on top of the conical tube with a Pasteur pipette and discard. Continue to remove and discard the remaining supernatant. For problems resulting from contamination of sample with fat, refer to troubleshooting 4).

**CRITICAL:** Take care not to disturb cell pellets at this stage as they may be loose (do not pour off supernatant).

**Figure 7:**
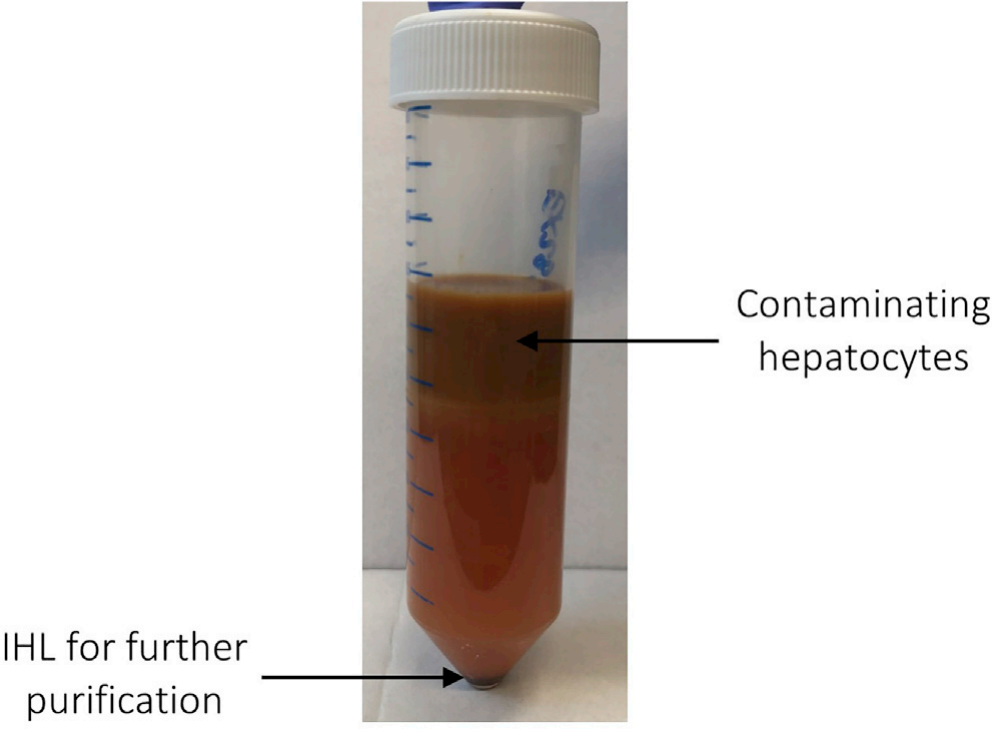
A representative example of the top layer of contaminating hepatocytes to be removed after centrifugation of 30% Percoll®-IHL solution (**steps 15 and 16**) The top layer of hepatocytes is removed and discarded prior to downstream purification of IHL from the cell pellet.

14. Vortex the remaining cell pellet and resuspend in 20 mL 30% Percoll® (v/v) in a 50 mL conical tube.

15. Centrifuge the Percoll®-IHL solution at 800 g using a swing bucket rotor for 15 min at R.T. (acceleration/deceleration: 9/9).
16. Remove the top layer of contaminating hepatocytes using a sterile Pasteur pipette and discard (refer to Figure 7). Continue to gently remove the remaining supernatant without disturbing the cell pellet. For difficulty removing the hepatocyte layer, refer to troubleshooting 5.

**CRITICAL:** Take care not to disturb cell pellets as they may be loose (do not pour off supernatant).

***Note:*** Due to the mechanical disruption, the hepatocytes at this stage are non-viable and, therefore, cannot be isolated in parallel to IHL using this protocol.

17. Vortex the cell pellet and resuspend in 25 mL of 1× HBSS^−/−^.

***Note:*** At this stage, cell pellets can be combined into 50 mL conical tubes before resuspending in 25 mL of 1× HBSS^−/−^ solution.

### Isolation of intrahepatic leukocytes (IHL)

**Timing: 1 h**

**Figure 8:**
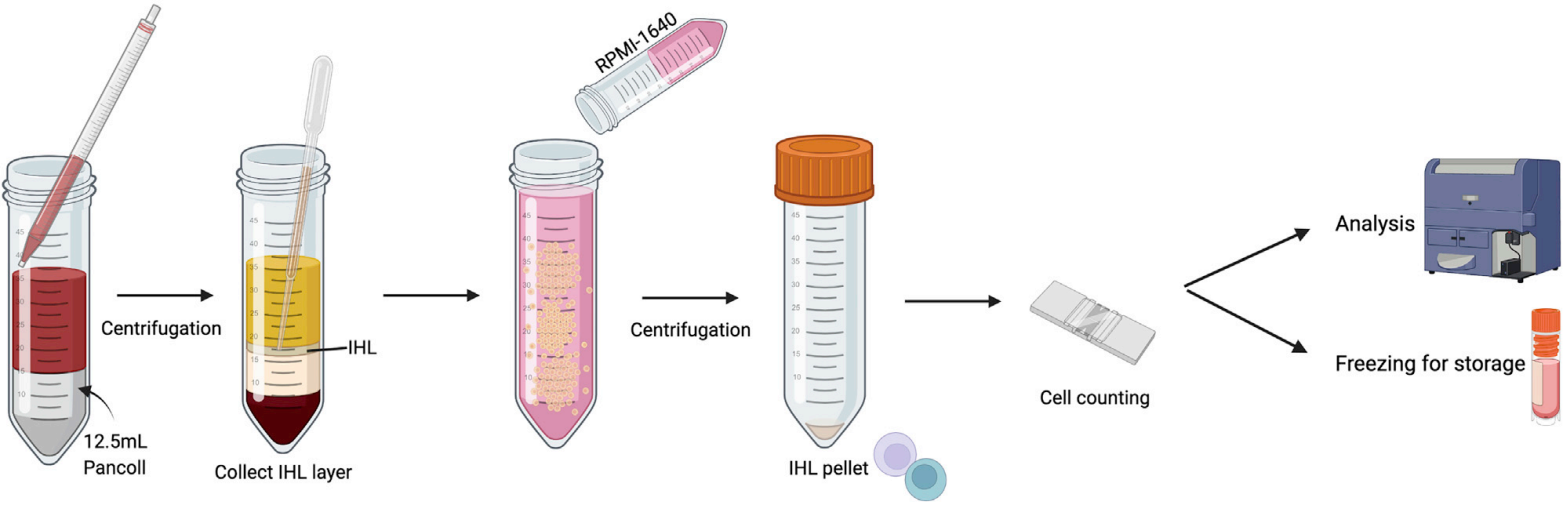
Illustration of the final steps required to isolate viable IHL from human tissue samples after the mechanical and enzymatic digestion of liver tissue and hepatocyte removal using a 30% Percoll gradient

The following steps provide information on how to isolate IHL suitable for short-term primary cell culture or *ex vivo* phenotypic and functional characterization by flow cytometry (Figure 8).

18. Gently layer 25 mL of the IHL single-cell suspension over 12.5 mL of Pancoll in a new 50 mL conical tube.

***Note:*** Ensure pipetting is very slow and care is taken to not disturb the layers.

19. Centrifuge the layered samples at 800 g using a swing bucket rotor for 20 min at R.T. (acceleration/deceleration: low / OFF).

**CRITICAL:** To maintain delicate layering of cells, ensure centrifuge acceleration and deceleration is set to low / OFF.

20. Harvest the IHL layer at the interface of the 1× HBSS^−/−^ and Pancoll gradient, using a Pasteur pipette, and transfer to a new 50 mL conical tube (refer to Figure 9 to identify layer of interest).

**Figure 9:**
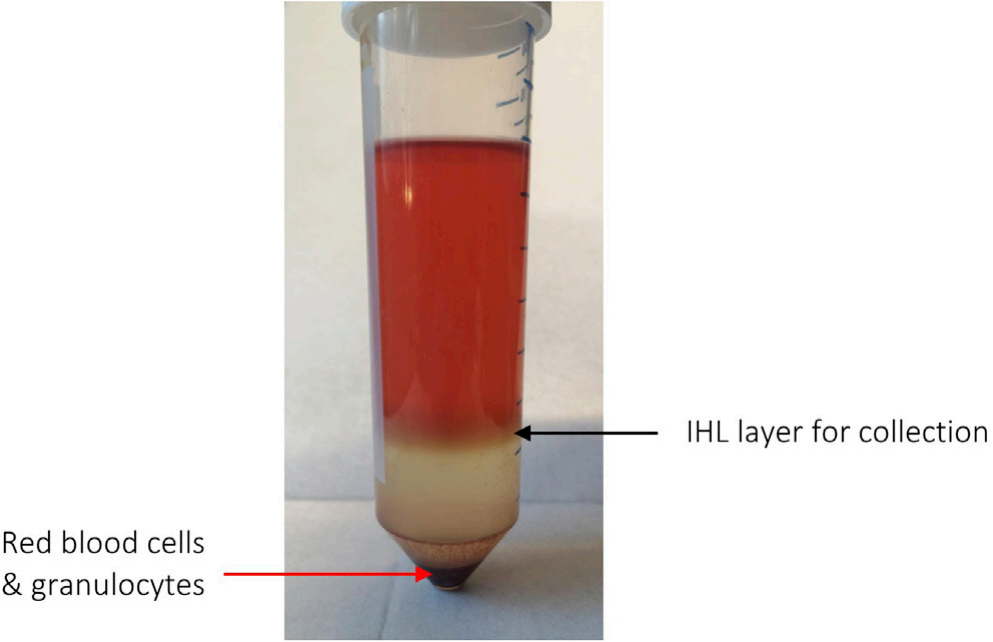
A representative example of the IHL cell layer after density centrifugation using a Pancoll gradient (from **step 19**) The layer indicated by the black arrow contains the IHL and is collected using a clean Pasteur pipette, prior to further processing (**step 20**).
21. Once carefully removed, dilute the IHL in RPMI-1640 to a final volume of 50 mL.
22. Centrifuge the diluted IHL solution at 700 g using swing buckets for 15 min at R.T. (acceleration/deceleration: 9/9).
23. Aspirate and discard the remaining supernatant with a 25 mL serological pipette without disturbing the cell pellet.
24. Resuspend the IHL pellet in an appropriate volume of complete RPMI-1640 for cell counting (for poor recovery refer to troubleshooting 1 and 2).

***Optional:*** If the IHL suspension is heavily contaminated with red blood cells (RBC), an RBC lysis step can be performed (refer to troubleshooting 5).

***Note:*** Isolated IHL are best counted with an automated cell counter to prevent inclusion of cellular debris. IHL can also be counted by eye with a haemocytometer and using trypan blue dye to differentiate dead cells.

**Pause point:** Following resuspension of the pellet, samples may be kept on ice or at 4°C. Alternatively IHL can be frozen down.

25. Purified IHL can be used immediately for culture or downstream analysis, or can be frozen for long-term storage. For freezing, centrifuge the isolated IHL suspension (700 g, 15 min, R.T.) and resuspend the resulting pellet in freezing media (refer to materials and equipment section) and aliquot into cryovials. For optimal cell recovery, place cryovials into a Mr.Frosty^TM^ freezing container in the −80°C freezer and transfer to liquid nitrogen after 24 h for long-term storage.

***Note:*** For optimal freezing, we recommend a minimum concentration of 5 × 10^6^ cells per 500 μL of freezing media and a maximum of 20 × 10^6^ cells per 1 mL of freezing media. Mr.Frosty^TM^ ensures a temperature drop of 1°C per minute, minimizing the risk of intracellular ice crystal formation and cell death.

### Staining of leukocytes for flow cytometry

The following steps provide information on how to examine IHL for phenotype and functional characterization by multi-parameter flow cytometry.

**CRITICAL:** Switch on flow cytometer before beginning staining protocol to allow lasers to warm up and equilibrate for a minimum of 20 min before use.

***Note:*** If performing this analysis from frozen samples, we recommend thawing IHL using pre-warmed 1× HBSS^+/+^ media supplemented with 0.002% DNase I to remove remaining DMSO. Thaw each vial in 20–25 mL of the DNase I supplemented 1× HBSS^+/+^ media and incubate at 37°C for 10–15 min before centrifuging at 700 g for 10 min at R.T. (acceleration/deceleration: 9/9). Discard supernatant and resuspend the pellet in DNase I supplemented 1× HBSS^+/+^. Further incubate for 10 min at 37°C prior to counting and analysis. Otherwise, proceed with the fresh samples from **step 25**.

26. Pre-cool centrifuge to 4°C.
27. Prior to starting staining, prepare LIVE/DEAD^TM^ Fixable Dead Cell Stain solution in 1× PBS at a suitable concentration – we recommend a dilution factor of 1 in 1,000.
28. Now prepare a master mix of fluorochrome-labeled antibodies against markers of interest using an appropriate panel of labeled-antibodies (refer to Table 1 for an example panel - the antibody dilutions in this table are titrated for staining in a volume of 50 μL). The total volume of master mix required will depend on number of samples being stained.

**Table 1:** Example antibody dilutions used in master mix preparation for detection of lymphocyte subsets from isolated IHL

Antigen	Fluorochrome	Dilution
CD45	BUV805	1:100
CD3	PE-Cy7	1:100
CD56	PE-Dazzle 549	1:100
CD19	BV786	2:100

**CRITICAL:** Antibody master mixes should be prepared in a 50:50 mix of 1× PBS and BD Brilliant Stain Buffer and protected from light. The use of BD Brilliant Stain Buffer is essential to prevent the generation of artifacts from interactions between dyes. The Brilliant Stain Buffer is not needed when only one brilliant dye is being used in the antibody master mix.

***Note:*** The LIVE/DEAD^TM^ Fixable Dead Cell Stain can be directly added to the antibody master mix to combine staining steps and reduce time needed for staining. However, we observed better separation of live and dead cells when stains are done separately, so ensure the optimization of the LIVE/DEAD^TM^ Stain concentration is carried out for detection of dead cells.

29. Transfer IHL in a maximum volume of 200 μL per well to a 96 well U-bottom tissue culture treated plate.

***Note:*** We recommend staining a maximum of 2 × 10^6^ cells per well of a 96 well U-bottom plate.

30. Centrifuge the plate at 500 g at 4°C for 4 min and gently discard the supernatant, careful not to disturb the cell pellet.
31. Resuspend cells in 50 μL of LIVE/DEAD^TM^ Stain solution per well. Incubate for either 15 min at R.T. or 30 min at 4°C protected from light.
32. Supplement each well with 150 μL 1× PBS and centrifuge at 500 g at 4°C for 4 min.
  ***Optional:*** Prior to staining cells with surface antibodies, you may wish to protect samples from non-specific antibody binding using 25 μL/well of FcR Block, prepared as per manufacturer’s instructions. This is particularly important if you require optimal staining of B cells, as represented in this example antibody panel. Following a 10 min incubation at 4°C, you may proceed directly to **step 33.**
33. Gently discard the supernatant and resuspend cells in 50 μL of your antibody master mix prepared in **step 27.** Leave cells to incubate for 30 min at 4°C protected from light.
34. Supplement each well with 150 μL 1× PBS and centrifuge at 500 g at 4°C for 4 min.
35. Gently discard the supernatant and resuspend the cell pellet in 100 μL BD Cytofix^TM^ buffer per well. Pipette up and down several times to avoid cell clumping. Incubate for 20 min at 4°C protected from light.

***Note:*** Alternative commercially available fixation solutions can be used, e.g., 1%–4% formaldehyde.

36. Supplement each well with 100 μL 1× PBS and centrifuge at 500 g at 4°C for 4 min.
37. Gently discard the supernatant and resuspend the cell pellet in 200 μL 1× PBS ready for acquisition by flow cytometry, transferring fully stained IHL into FACS tubes.

**Pause point:** Stained samples may be run the following day when kept at 4°C and protected from light. To avoid the degradation of the fluorochromes, we recommend the analysis be carried out as soon as possible.

***Note:*** We recommend placing a 1.2 mL ‘small insert’ tube (e.g., Corning 96-well Cluster Tubes, Cat# 4401) with the stained cells inside a FACS tube to reduce the residual volume, which is important when working with limiting cell numbers. If the flow cytometer model allows, samples may also be analyzed using a HTS sample coupler for acquisition of samples from a 96-well plate.

38. Acquire data using an appropriate flow cytometer (see materials and equipment section on 5-laser BD LSRFortessa X20 flow cytometer for configuration) running BD FACSDiva v. 9.0.1.
39. Analyze data using appropriate software, such as FlowJo v. 10.

***Note:*** For more complex staining, including detection of intracellular or intranuclear molecules, refer to our staining protocol (Cossarizza et al., 2021). For assessment of cellular functionality and the detection of intracellular molecules such as cytokines and chemokines, an appropriate stimulus, and the use of protein transport inhibitors (Monensin and/or Brefeldin A) will often be required.

## Expected outcomes

### Expected IHL cell yield per gram of tissue processed

**Figure 10:**
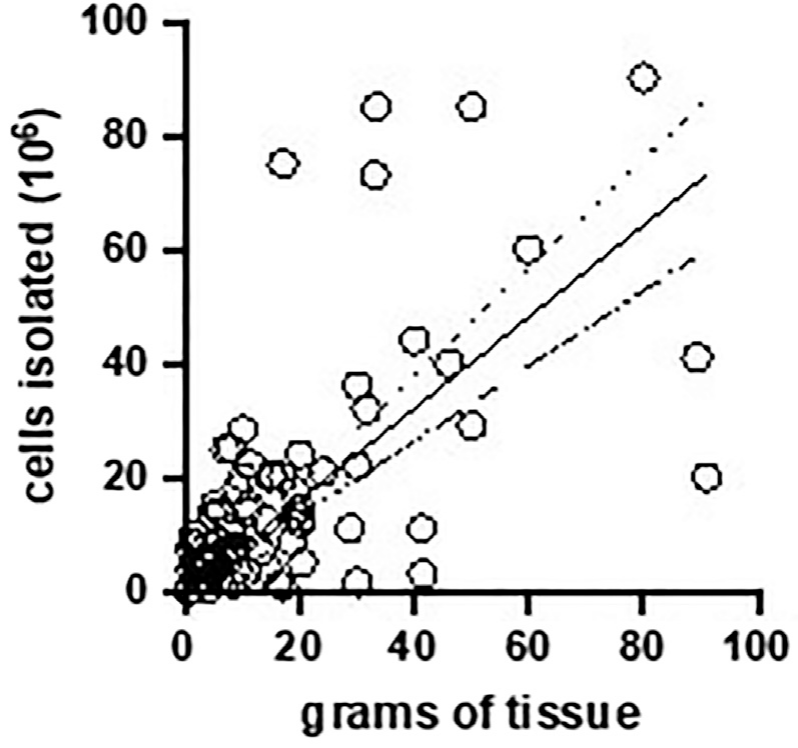
Graph presenting the linear relationship between the mass of liver tissue processed vs. total number of live intrahepatic leukocytes isolated using our optimized protocol (R^2^ = 0.71; p<0.0001), for all samples analyzed (n=104)

The protocol developed and described here allows for the consistent and reproducible isolation of IHL of high viability and purity from varying sized samples, including larger volume tissue samples typically obtained from resection or explant surgeries. This protocol has been used to process over 160 samples and is scalable in terms of the expected IHL cell yield and amount of liver processed: Figure 10 demonstrates the linear relationship observed (R^2^ = 0.71; p<0.0001) between the weight (in grams) of tissue processed and the number of IHL obtained.

Using this protocol, you can expect a mean yield of ∼1.04 × 10^6^ IHL (± 0.90 S.D.) per gram of tissue processed, with a mean viability of 91% (as determined by flow cytometric assessment; refer to Table 2). However, IHL yield can be significantly influenced by the quality of the tissue sample and the underlying disease etiology associated with the sample, as determined by an independent histopathologist (refer to Table 2 for a breakdown by underlying sample histology). When processing highly fibrotic or cirrhotic tissue samples marked rigidity is often noted, likely arising from increased extracellular matrix deposition, and a significant drop in cell yield is seen (Table 2).

**Table 2:** Expected IHL yield and viability by tissue source

Tissue source	Expected cell yield/gram of tissue processed; # cells × 10^6^ IHL/g (mean ± SD)	Expected lymphocyte viability as determined by flow cytometry; %CD45^+^ live lymphocytes (mean ± SD)
All liver samples	1.04 ± 0.90	91.39 ± 11.60
Histologically normal tissue	1.37 ± 1.03	92.02 ± 8.27
Histologically fibrotic tissue	0.64 ± 0.49	85.95 ± 21.79
Histologically cirrhotic tissue	0.58 ± 0.34	90.17 ± 11.43
Primary liver tumor samples (HCC TILs)	1.7 ± 3.92	73.09 ± 25.22

SD = standard deviation.

Please note: This protocol is also suitable for the isolation of paired tumor-infiltrating leukocytes (TIL) from liver tumors taken from the same individual, with an average cell yield (per gram of tissue) comparable to that of the background non-tumor associated liver tissue but with a lower mean viability (refer to Table 2). However, in our experience processing paired TIL samples, a wide range can be expected in terms of cell yield, which may reflect biological differences between tumor types.

### Intrahepatic immune cell subset distribution

**Figure 11:**
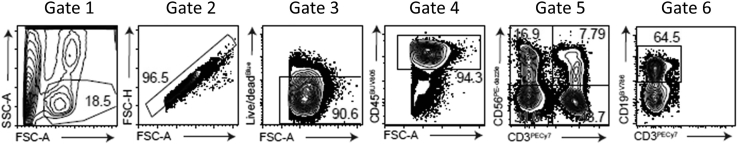
Gating strategy showing the analysis of viable human lymphocyte subsets from isolated IHL sampled from liver resections and explants Lymphocytes are discriminated by size by plotting FSC-A against SSC-A (Gate 1). Single cells are identified by plotting FSC-A against FSC-H (Gate 2). Live cells are defined as LIVE/DEAD^TM^ Fixable Blue Dead Cell stain negative (Gate 3). Pan-leukocytes are further defined as CD45^+^ (Gate 4). Plotting CD3 expression against CD56 expression is used to identify CD3^+^CD56^-^ T cells, CD3^+^CD56^+^ NKT cells and CD3^-^CD56^+^ NK cells (Gate 5). CD3^-^CD56^-^ cells are further gated to identify CD19^+^ B cells (Gate 6).

Further to revealing an expected IHL cell yield we can provide some basic information on the expected intrahepatic immune cell subset composition as determined by flow cytometry. Figure 11 shows a representative gating strategy used to differentiate B cells, T cells, NK cells and NKT cells with their expected average cell frequencies presented in Table 3 by histological status (not taking into account any disease specific differences).

**Table 3:** Intrahepatic immune cell subset frequencies (of CD45^+^ live cells) by liver tissue source

Tissue source	CD3^+^ T cells (% CD45^+^ cells) (Mean)	CD19^+^ B cells (% CD45^+^ cells) (Mean)	CD3^-^ CD56^+^ NK cells (% CD45^+^ cells) (Mean)	CD3^+^ CD56^+^ NKT cells (% CD45^+^ cells) (Mean)	Other (% CD45^+^ cells) (Mean)
Normal liver	25.45	7.49	38.95	19.87	8.24
Fibrotic liver	35.34	12.09	33.32	13.05	6.2
Cirrhotic liver	36.47	13.05	33.9	8.22	8.36

For further information on expected frequencies and phenotypic marker expression refer to the following published research articles that have used this protocol (Easom et al., 2018; Pallett et al., 2017, 2020; Stegmann et al., 2016; Swadling et al., 2020; Zakeri et al., 2022).

## Limitations

Mechanical dissociation of tissue, although widely used in cell isolation protocols, has previously been associated with reduced cell viability and purity. Conversely, it is widely accepted that enzymatic digestion of tissue helps to increase cell yield, perhaps at the expense of cleavage of some cell surface proteins. However, by stringently following the timings and concentration of enzymes listed in this protocol, we have shown that it is possible to navigate these limitations to obtain samples of high quality for both functional and phenotypic characterization. The use of both a Percoll® step to first remove contaminating hepatocytes and the subsequent Pancoll (or similar) density gradient purification step, allows for the isolation of IHL sample of high yield and purity. However, it must be noted that these additional steps increase processing time and the potential risk of increased cell loss at each stage during centrifugation. Where contaminating cellular material is present, this may result in excess non-specific antibody binding and background autofluorescence of the sample, which can be particularly problematic when discriminating between multiple discrete immune cell populations by flow cytometry analysis.

## Troubleshooting

### Problem 1

Tissue accessibility and delays relating to sample collection.

### Potential solution

Due to delays in surgery timings and the availability of qualified histopathologists to dissect tissue deemed surplus to diagnostic requirements, tissue processing might have to be performed several hours after the initial surgical removal from the patient. Our protocol has been used to successfully isolate IHL from samples processed after a range of ‘waiting’ times and importantly we did not observe a significant decrease in the expected IHL yield if processed more than 6 h post-surgery (refer to Table 4), in line with published literature (Curry et al., 2000; Kinoshita et al., 1998). However, we note a slight trend toward a decreased isolation efficiency between the two timepoints tested using this protocol, which leads us to recommend a maximum of 24 h delay in processing. We recommend keeping the liver tissue samples refrigerated in UW solution or RPMI-1640 media prior to processing (if a delay is unavoidable) to help with IHL recovery.

**Table 4:** Impact of delayed liver tissue processing on lymphocyte isolation and viability

	Expected cell yield/gram of tissue processed; # cells × 10^6^ IHL/g (mean ± SD)	Expected lymphocyte viability as determined by flow cytometry; %CD45^+^ live lymphocytes (mean ± SD)
Processed within 6 h	1.14 ± 0.84	93.39 ± 8.59
Processed after 6 h	1.02 ± 1.21	89.73 ± 13.20

SD = standard deviation.

### Problem 2

Low IHL yield or poor viability - impact of enzymatic digestion on cell retrieval and quality.

### Potential solution

Low IHL yield or poor viability is associated with a poor isolation technique. Prior to commencing, we suggest users ensure all reagents and equipment are prepared in advance to the appropriate volume, concentration and temperature as described in this protocol. When processing large or multiple samples, we suggest staggering the protocol where necessary to ensure all timings are adhered to (**steps 6–11**). Prolonged incubation with digestion enzymes may lead to the loss of cell surface markers; however, we confirm no loss of expression of CD56, CD19 and CD3 (data not shown), in line with published studies showing no loss of expression of key markers when specific incubation periods are adhered to (Li et al., 2013; Morsy et al., 2005; Shi et al., 2020). Insufficient digestion time can lead to inadequate liberation of cells from tissue, resulting in a poorer IHL yield. When adhering to the stated enzyme concentrations and timings outlined here, we can confirm an improved IHL isolation efficiency with combined mechanical and enzymatic digestion compared to mechanical dissociation alone using a small subset of our samples (refer to Table 5). Furthermore, to not compromise IHL yield or viability, it is important that at no stage the liver be left dry, particularly during the dissecting (**step 2**) and grinding stages (**step 11**). To avoid this, be sure to dissect tissue in solution and to continually rinse the cell strainer with media such as 1× HBSS^−/−^.

**Table 5:** Comparison between tissue dissociation methods with and without the use of enzymes in a targeted paired cohort

	Expected cell yield/gram of tissue processed; # cells × 10^6^ IHL/g (mean ± SD)	Expected lymphocyte viability as determined by flow cytometry; %CD45^+^ live lymphocytes (mean ± SD)
Enzymatic and mechanical dissociation	0.86 ± 0.49	82.12 ± 14.60
Mechanical dissociation	0.67 ± 0.62	81.95 ± 13.62

SD = standard deviation.

### Problem 3

Difficulty passing liver tissue through a 70 μm cell strainer (**step 9**).

### Potential solution

Liver tissue samples independently characterized as histologically fibrotic or cirrhotic are more difficult to process than samples classified as histologically normal. Fibrotic and cirrhotic tissue is marked by increased rigidity and loss of liver architecture and can be notably harder to pass through a 70 μm cell strainer. This can be partially mitigated by carefully removing fibrotic plaques/tissue while dissecting the liver tissue using blunt-ended scissors to approximately <5 mm^3^ pieces. We suggest further mechanically disrupting such tissue samples by running an additional 2–3 cycles on the gentleMACS^TM^ tissue dissociator (*m_spleen_01.01*; **step 7**) prior to grinding small amounts through each cell strainer using the flat end of a syringe plunger with plenty of media. Where necessary, remove chunks of hard fibrous tissue and regularly change the cell strainer if it becomes clogged.

### Problem 4

Fatty liver tissue samples – disrupting density centrifugation steps.

### Potential solution

Liver tissue samples with a high fat content can become apparent during processing, particularly after centrifugation at **step 16**. This will disturb density based gradient centrifugation due to its lower density rendering IHL isolation less optimal. To successfully isolate leukocytes without excessive cell loss or contamination of sample, it is essential to remove the fatty layer illustrated in Figure 6 prior to density centrifugation steps. This can be achieved by gently aspirating and discarding the fat layer using a sterile plastic Pasteur pipette. Transfer pellets to new tubes for density centrifugation.

### Problem 5

Contamination of IHL with hepatocytes and/or red blood cells (**steps 16 and 24**).

### Potential solution

After Percoll density gradient separation the layer of hepatocytes concentrated at the top must be delicately removed using a Pasteur pipette. If the IHL solution is highly concentrated due to insufficient dilution in media post grinding, evidenced by a cloudy supernatant, an additional washing step may be required followed by a second Percoll density gradient separation using a fresh conical tube. In addition, if the final IHL pellet is heavily stained red (indicating red blood cell [RBC] contamination), this may require treatment with RBC lysis buffer as per manufacturer’s instructions (refer to key resources table and alternative resources table) prior to proceeding to **step 25** depending on your downstream experimental needs.

## Resource availability

### Lead contact

Further information and requests for resources and reagents should be directed to and will be fulfilled by the lead contact Dr. Laura J. Pallett, laura.pallett@ucl.ac.uk.

### Materials availability

This study did not generate new unique reagents.

## Data Availability

The data used in support of the generation and optimization of this study have not been deposited in a public repository but are available from the corresponding author upon reasonable request.
